# Supplementary material for: Rational Design of a Low-Data Regime of Pyrrole Antioxidants for Radical Scavenging Activities Using Quantum Chemical Descriptors and QSAR with the GA-MLR and ANN Concepts
Source: Molecules. 2023 Feb 7;28(4):1596. doi: 10.3390/molecules28041596 (PMC9959680; doi:10.3390/molecules28041596)
Supplement: Supplementary file 1 [file molecules-28-01596-s001.zip › molecules-2191099-supplementary.pdf]

Supporting Information:

Rational Design of a Low-Data Regime of Pyrrole Antioxidants for Radical Scavenging Activities Using Quantum Chemical Descriptors and QSAR with the GA-MLR and ANN Concepts

Wanting Xie [1], Sopon Wiriyarattanakul [2], Thanyada Rungrotmongkol [3,4], Liyi Shi [1,5], Amphawan Wiriyarattanakul [6,*], and Phornphimon Maitarad [1,*]

[1] Research Center of Nano Science and Technology, College of Sciences, Shanghai University, Shanghai 200444, China; wantingshieh@shu.edu.cn (W.X.); shiliyi@shu.edu.cn (L.S.)
[2] Program in Computer Science, Faculty of Science and Technology, Uttaradit Rajabhat University, Uttaradit 53000, Thailand; sopon@uru.ac.th
[3] Center of Excellence in Structural and Computational Biology, Department of Biochemistry, Chulalongkorn University, Bangkok 10330, Thailand; t.rungrotmongkol@gmail.com
[4] Program in Bioinformatics and Computational Biology, Graduate School, Chulalongkorn University, Bangkok 10330, Thailand
[5] Emerging Industries Institute, Shanghai University, Jiaxing, Zhejiang, 314006 China
[6] Program in Chemistry, Faculty of Science and Technology, Uttaradit Rajabhat University, Uttaradit 53000, Thailand
* Correspondence: amphawan_m@uru.ac.th (A.W.); pmaitarad@shu.edu.cn (P.M.)

List of supporting information

No	Figure/Table	Page
**1**	**Table S1**. Experimental and predicted radical scavenging activities of 15 studied compounds from QSAR GA-MLR and ANN models.	**P3**
**2**	**Table S2.** 33 molecular properties of 15 pyrrole derivatives.	**P4**
**3**	**Table S3.** 33 molecular properties of 17 new designed pyrrole derivatives.	**P6**
**4**	**Table S4.** 17 new designed compounds structures and their predicted radical scavenging activities from QSAR GA-MLR and ANN models.	**P8**
**5**	**Figure S1**. Experimental activities of previous synthesized compounds (1-15) and predicted antioxidant activities of new designed compounds (16-32) by ANN models.	**P11**
**6**	**Figure S2.** Starting reagents with codes and pyrrole derivatives' skeleton diagram with atom labels.	**P12**
**7**	**Table S5.** 15 Pyrrole derivatives and their radical scavenging activities.	**P13**

Table S1. Experimental and Predicted radical scavenging activities of 15 studied compounds from QSAR GA-MLR (normal letters) and ANN (Italic letters) models. Compounds with a star mark are the test subset for model.

**Cpd.**	**·OH**			**$O_2^{·-}$**			**DPPH·**		
	**Exp.**	**Pred.**	***Pred.***	**Exp.**	**Pred.**	***Pred.***	**Exp.**	**Pred.**	***Pred.***
**1**	9.047	9.183	*9.121*	7.811	7.811	*7.793*	4.627	5.229	*5.059*
**2**	8.01	7.917	*7.894*	7.622	7.789	*7.776*	7.126	6.478	*6.610*
**3**	8.991	9.183	*9.087*	8.644	8.117	*8.215*	5.517	4.486	*5.242*
**4**	6.848	6.763	*6.653*	6.781	7.005	*6.879*	1.772	2.280	*2.307*
**5**	7.588	7.525	*7.438*	6.485	6.773	*6.652*	1.561	2.975	*2.095*
**6***	6.365	6.597	*6.629*	6.498	6.243	*6.397*	2.504	1.755	*1.948*
**7**	6.658	7.244	*6.970*	6.341	6.132	*6.315*	7.604	6.132	*7.545*
**8***	7.094	7.420	*6.860*	6.443	6.941	*6.839*	3.211	3.248	*4.119*
**9**	7.388	7.836	*7.712*	6.578	6.977	*6.808*	1.747	2.361	*1.585*
**10**	6.681	6.295	*6.645*	6.371	6.120	*6.358*	3.889	2.771	*3.531*
**11***	7.294	6.943	*6.708*	6.203	6.138	*6.343*	1.348	2.069	*1.138*
**12**	9.151	8.499	*8.980*	8.057	8.055	*8.147*	7.044	6.730	*6.719*
**13**	6.757	7.202	*6.858*	7.209	6.903	*6.769*	6.391	7.721	*6.957*
**14**	8.148	7.830	*8.069*	7.832	7.807	*7.842*	4.745	4.179	*4.179*
**15**	8.431	8.223	*8.275*	7.264	7.505	*7.416*	6.941	7.622	*7.088*

Table S2. 33 molecular properties of 15 pyrrole derivatives.

Cpd.	[a] X1	X2	X3	X4	X5	X6	X7	X8	X9	X10	X11	X12	X13	X14	X15	X16	X17	X18	X19
**1**	0.351	-0.364	0.413	0.008	0.173	-0.198	0.019	-0.592	0.074	-0.487	-0.347	-0.585	-0.646	-0.184	0.245	1.43	1.485	1.486	1.448
**2**	0.119	-0.359	0.428	0.195	0.578	-0.323	-0.298	-0.577	0.115	-0.507	-0.199	-0.581	-0.249	1.198	0.079	1.459	1.488	1.487	1.448
**3**	0.343	-0.049	0.4	-0.069	0.636	-0.307	-0.155	-0.573	0.026	-0.519	-0.059	-0.572	-1.135	-0.364	0.126	1.43	1.475	1.486	1.451
**4**	0.237	-0.479	0.605	0.037	0.488	-0.604	-0.105	-0.491	0.398	-0.487	0.096	-0.418	-1.067	-0.159	0.145	1.424	1.486	1.482	1.48
**5**	0.139	-0.319	0.695	-0.388	0.399	0.089	0.015	-0.488	0.322	-0.487	-0.307	-0.345	-0.248	0.208	0.206	1.421	1.487	1.481	1.482
**6**	0.192	-0.225	0.411	-0.007	0.532	-0.29	0.009	-0.493	-0.439	-0.473	0.056	-0.441	-0.675	-0.185	0.199	1.425	1.485	1.483	1.479
**7**	0.131	0.057	0.771	-0.117	0.269	-0.012	0.027	-0.497	-0.803	-0.415	-0.831	-0.383	-0.452	-0.017	0.073	1.449	1.487	1.483	1.484
**8**	0.141	-0.288	0.269	0.023	0.351	-0.215	0.098	-0.492	-0.54	-0.461	0.159	-0.457	-0.203	0.16	0.023	1.425	1.473	1.482	1.481
**9**	0.29	0.012	0.357	-0.254	0.566	-0.382	0.263	-0.508	-0.535	-0.471	0.09	-0.507	-1.137	-0.482	0.206	1.427	1.483	1.483	1.463
**10**	0.157	-0.516	0.352	-0.077	0.298	-0.196	0.263	-0.526	-0.2	-0.474	0.25	-0.468	-0.217	0.248	0.144	1.42	1.486	1.482	1.479
**11**	0.284	-0.137	0.941	-0.236	0.386	-0.183	0.063	-0.545	-0.226	-0.462	-0.706	-0.383	-1.11	-0.182	0.114	1.427	1.484	1.483	1.484
**12**	0.251	-0.166	0.255	-0.27	0.271	-0.39	0.196	-0.501	-0.337	-0.44	-0.132	-0.473	-0.506	-0.202	0.267	1.429	1.485	1.484	1.464
**13**	0.185	-0.343	0.547	-0.039	0.47	-0.001	0.171	-0.565	-0.598	-0.41	0.209	-0.44	-0.245	0.083	0.052	1.452	1.486	1.48	1.478
**14**	0.173	-0.337	1.309	-0.06	0.135	-0.18	0.074	-0.553	-0.374	-0.437	-1.048	-0.336	-0.246	0.058	0.194	1.425	1.485	1.482	1.483
**15**	0.057	-0.395	0.556	0.039	0.37	0.035	0.068	-0.57	-0.539	-0.404	-0.114	-0.501	0.024	0.331	0.08	1.46	1.475	1.479	1.462

Cpd.	X20	X21	X22	X23	[b]X24	X25	X26	X27	X28	X29	X30	X31	X32	X33
**1**	378.364	-0.288	0.054	0.342	4	5	4.535	8.478	128.856	5.991	480.819	494.969	789.41	1400
**2**	323.387	-0.292	0.054	0.346	7	5	2.753	6.115	126.443	6.041	428.505	432.877	718.27	1250
**3**	333.973	-0.297	0.055	0.352	4	5	2.396	6.537	123.797	4.953	415.767	417.595	696.532	1210
**4**	331.347	-0.3	0.053	0.352	5	6	4.071	8.486	106.429	5.63	420.06	428.381	701.071	1230
**5**	395.19	-0.293	0.053	0.346	6	5	6.022	11.868	153.052	6.465	482.605	503.4	787.087	1410
**6**	352.486	-0.295	0.033	0.328	5	4	4.228	6.072	103.493	5.697	413.108	435.068	676.949	1210
**7**	313.295	-0.29	0.054	0.344	8	4	5.286	8.191	151.474	7.215	492.748	529.654	788.413	1450
**8**	404.09	-0.307	0.022	0.329	5	6	2.926	4.824	96.271	5.735	410.652	431.972	672.939	1210
**9**	377.594	-0.287	0.056	0.343	4	4	4.955	7.064	118.593	5.617	439.343	458.497	719.282	1290
**10**	303.826	-0.301	0.054	0.355	6	5	3.032	6.081	122.247	6.154	409.979	425.097	674.031	1200
**11**	307.325	-0.298	0.03	0.328	6	4	5.281	7.107	121.85	6.246	441.895	456.261	722.997	1290
**12**	333.568	-0.289	0.054	0.343	5	5	3.805	8.114	151.122	6.056	492.696	509.598	804.94	1440

Cpd.	X20	X21	X22	X23	[b] X24	X25	X26	X27	X28	X29	X30	X31	X32	X33
**13**	302.371	-0.29	0.056	0.346	7	5	2.313	6.196	139.041	6.293	449.603	464.679	746.933	1320
**14**	345.037	-0.289	0.053	0.342	6	7	5.932	9.742	122.01	6.987	499.443	511.825	815.299	1450
**15**	383.613	-0.3	0.038	0.339	6	5	0.801	5.143	117.561	5.272	402.967	413.541	668.653	1180

[a] Properties **X1~X23**: Charge of N1, Charge of C2, Charge of C3, Charge of C4, Charge of C5, Charge of C6, Charge of C7, Charge of O8, Charge of C9, Charge of O10, Charge of C11, Charge of O12, Charge of C13, Total Charge R(d), Total charge R(b), Bond N1-C13, Bond C2- R(b), Bond C3-C6, Bond C4-C11, Polarizability, HOMO Energy, LUMO Energy, Energy Gap.(quantum chemistry properties obtained from HF/6-31G(d,p) functional.)

[b] Properties **X24~X33**: Rotatable bonds , Hydrogen bond acceptor , AlogP , AlogP98, Molecular refractivity, Molecular flexibility, Connolly surface area, Connolly surface occupied volume, Solvent surface area, Solvent surface occupied volume.(Molecular properties calculated via QSAR model module in Materials Studio program)

X1	Charge of N1	X12	Charge of O12	X23	Energy Gap
X2	Charge of C2	X13	Charge of C13	X24	Rotatable bonds
X3	Charge of C3	X14	Total Charge R(d)	X25	Hydrogen bond acceptor
X4	Charge of C4	X15	Total charge R(b)	X26	AlogP
X5	Charge of C5	X16	Bond N1-C13	X27	AlogP98
X6	Charge of C6	X17	Bond C2-R(b)	X28	Molecular refractivity
X7	Charge of C7	X18	Bond C3-C6	X29	Molecular flexibility
X8	Charge of O8	X19	Bond C4-C11	X30	Connolly surface area
X9	Charge of C9	X20	Polarizability	X31	Connolly surface occupied volume
X10	Charge of O10	X21	HOMO Energy	X32	Solvent surface area
X11	Charge of C11	X22	LUMO Energy	X33	Solvent surface occupied volume

Table S3. 33 molecular properties of 17 new designed compounds.

Cpd.	[a] X1	X2	X3	X4	X5	X6	X7	X8	X9	X10	X11	X12	X13	X14	X15	X16	X17	X18	X19
**16**	0.272	-0.328	0.503	-0.062	0.143	-0.013	-0.024	-0.582	0.029	-0.518	-0.322	-0.572	-0.379	-0.044	0.128	1.430	1.475	1.486	1.448
**17**	0.079	-0.426	0.634	0.077	0.413	-0.313	-0.177	-0.575	0.165	-0.519	-0.481	-0.577	-0.261	0.26	-0.183	1.459	1.476	1.486	1.447
**18**	0.216	-0.369	0.561	0.048	0.174	-0.13	-0.275	-0.572	0.208	-0.52	-0.476	-0.578	-0.468	0.195	0.077	1.455	1.476	1.486	1.448
**19**	0.312	-0.118	0.396	0.014	0.508	-0.283	-0.173	-0.573	0.031	-0.518	-0.072	-0.573	-0.751	-0.169	0.163	1.430	1.475	1.486	1.451
**20**	0.084	-0.448	0.441	0.194	0.437	-0.227	-0.187	-0.578	0.228	-0.511	-0.303	-0.588	-0.454	0.358	0.14	1.462	1.475	1.486	1.449
**21**	0.237	-0.206	0.275	0.115	0.29	-0.335	-0.212	-0.569	0.259	-0.526	-0.309	-0.581	-0.433	0.242	0.08	1.455	1.476	1.486	1.451
**22**	0.316	-0.683	0.861	1.216	-0.8	-0.058	-0.172	-0.553	0.044	-0.513	-0.27	-0.516	-1.557	-0.352	0.165	1.429	1.475	1.487	1.472
**23**	0.117	-0.907	0.962	0.925	-0.895	-0.162	-0.084	-0.55	0.087	-0.522	-0.268	-0.513	-0.254	0.271	0.136	1.459	1.476	1.486	1.470
**24**	0.16	-0.137	0.902	0.564	-0.692	-0.243	-0.203	-0.549	0.052	-0.518	-0.485	-0.518	-0.44	0.275	0.154	1.461	1.475	1.486	1.471
**25**	0.246	-0.2	0.546	-0.059	0.277	-0.27	0.066	-0.564	-0.562	-0.42	-0.19	-0.487	-0.921	-0.256	0.177	1.428	1.472	1.480	1.461
**26**	0.053	-0.413	0.789	-0.07	0.312	-0.143	0.072	-0.571	-0.449	-0.417	-0.271	-0.493	-0.298	0.248	0.095	1.457	1.475	1.479	1.460
**27**	0.146	-0.167	0.643	-0.181	0.168	-0.007	0.026	-0.573	-0.439	-0.403	-0.28	-0.488	-0.448	0.179	0.186	1.452	1.475	1.479	1.461
**28**	0.233	-0.125	0.511	-0.215	0.529	-0.285	0.095	-0.564	-0.559	-0.421	0.174	-0.493	-0.81	-0.191	0.198	1.428	1.472	1.480	1.463
**29**	0.053	0.151	0.412	-0.136	0.526	-0.033	-0.11	-0.551	-0.499	-0.408	-0.088	-0.503	-0.215	0.158	-0.074	1.450	1.475	1.479	1.463
**30**	0.278	-0.475	0.971	1.036	-0.772	0.273	-0.088	-0.553	-0.361	-0.4	-0.502	-0.393	-1.206	-0.288	0.2	1.427	1.473	1.481	1.474
**31**	0.144	-0.869	1.149	0.992	-1.037	0.215	-0.021	-0.562	-0.412	-0.395	-0.577	-0.396	0.022	0.339	0.188	1.459	1.475	1.481	1.472
**32**	0.153	0.26	0.911	0.587	-0.742	-0.082	-0.311	-0.537	-0.209	-0.399	-0.854	-0.387	-0.203	0.259	-0.091	1.454	1.474	1.480	1.474

Cpd.	X20	X21	X22	X23	[b] X24	X25	X26	X27	X28	X29	X30	X31	X32	X33
16	358.960	-0.297	0.054	0.351	5	6	2.440	6.664	134.243	5.616	454.921	466.307	749.148	1330
17	323.649	-0.296	0.058	0.354	6	5	1.986	7.215	123.135	5.473	424.816	434.509	707.151	1240
18	353.587	-0.297	0.055	0.352	5	5	2.787	5.695	132.615	5.409	438.564	456.683	726.014	1290
19	337.158	-0.298	0.052	0.350	5	6	1.676	6.206	125.219	5.346	425.637	428.894	712.545	1240
20	300.156	-0.295	0.052	0.347	6	5	1.454	4.982	112.852	5.217	389.978	397.477	654.438	1140
21	331.708	-0.298	0.053	0.351	5	5	2.024	5.237	123.591	5.140	409.137	418.638	687.867	1200
22	373.914	-0.304	0.057	0.360	7	6	3.062	8.618	137.950	6.188	462.826	460.694	777.065	1340
23	337.426	-0.303	0.059	0.361	8	5	4.048	8.450	127.477	6.110	434.384	429.241	734.896	1260
24	367.814	-0.303	0.056	0.359	7	5	4.618	10.005	138.217	5.981	451.139	450.654	758.914	1310

Cpd.	X20	X21	X22	X23	X24	X25	X26	X27	X28	X29	X30	X31	X32	X33
25	365.758	-0.298	0.028	0.326	4	6	1.876	7.179	128.295	5.682	467.094	482.593	766.265	1360
26	329.897	-0.302	0.029	0.331	6	5	1.565	5.601	126.585	5.533	437.775	450.625	724.052	1280
27	360.082	-0.304	0.028	0.332	5	5	2.134	5.856	137.325	5.475	451.736	473.487	740.818	1320
28	344.301	-0.298	0.028	0.327	4	6	1.112	6.721	119.272	5.409	437.587	443.126	728.376	1280
29	337.551	-0.306	0.028	0.334	5	5	1.370	6.698	128.301	5.203	421.026	436.423	701.621	1240
30	381.842	-0.305	0.030	0.335	7	6	2.409	8.779	142.660	6.244	474.630	477.189	791.136	1380
31	343.724	-0.307	0.030	0.337	8	5	2.187	7.555	130.292	6.155	442.692	447.500	738.834	1290
32	374.340	-0.312	0.029	0.340	7	5	2.756	9.110	141.031	6.037	461.160	469.527	766.002	1340

[a] Properties **X1~X23**: Charge of N1, Charge of C2, Charge of C3, Charge of C4, Charge of C5, Charge of C6, Charge of C7, Charge of O8, Charge of C9, Charge of O10, Charge of C11, Charge of O12, Charge of C13, Total Charge of R(d), Total charge of R(b), Bond N1-C13, Bond C2- R(b), Bond C3-C6, Bond C4-C11, Polarizability, HOMO Energy, LUMO Energy, Energy Gap.(quantum chemistry properties obtained from HF/6-31G(d,p) functional.)

[b] Properties **X24~X33**: Rotatable bonds , Hydrogen bond acceptor , AlogP , AlogP98, Molecular refractivity, Molecular flexibility, Connolly surface area, Connolly surface occupied volume, Solvent surface area, Solvent surface occupied volume.(Molecular properties calculated via QSAR model module in Materials Studio program)

Table S4. 17 newly designed compounds structures and their predicted radical scavenging activities from QSAR GA-MLR (normal letters) and ANN (Italic letters) models.

Cpd.	Structure	Code	$^{\bullet}OH$ activity [a]	$O_2^{\bullet-}$ activity [b]	$DPPH^{\bullet}$ activity [c]
16	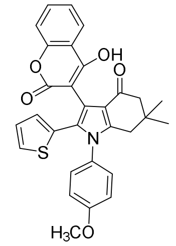	1a2c1b4a	9.702<br>*9.060*	8.337<br>*8.457*	6.475<br>*5.717*
17	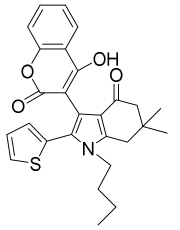	1a2c1b4b	9.088<br>*9.065*	8.114<br>*8.208*	7.255<br>*6.875*
18	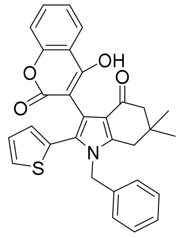	1a2c1b4g	9.545<br>*9.077*	8.339<br>*8.461*	6.728<br>*6.617*
19	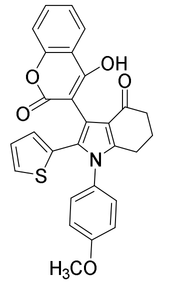	1a2c1c4a	9.231<br>*9.091*	8.195<br>*8.310*	5.873<br>*5.927*
20	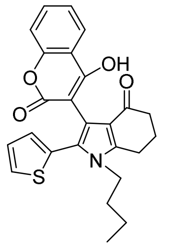	1a2c1c4b	8.669<br>*8.856*	7.848<br>*7.865*	6.234<br>*6.932*
21	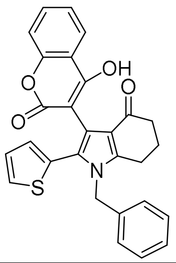	1a2c1c4g	9.076<br>*9.077*	8.196<br>*8.313*	6.126<br>*6.588*

22	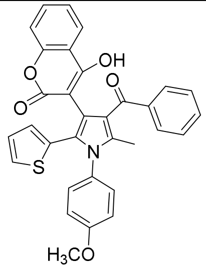	1a2c3b4a	8.809<br>*9.103*	7.881<br>*7.933*	6.042<br>*5.396*
23	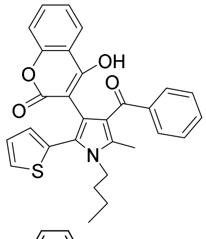	1a2c3b4b	8.221<br>*8.417*	7.705<br>*7.693*	5.158<br>*5.830*
24	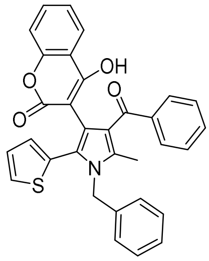	1a2c3b4g	8.728<br>*9.060*	7.849<br>*7.888*	5.456<br>*6.376*
25	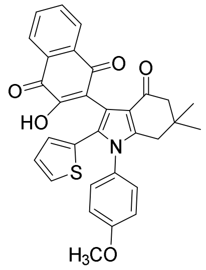	1d2c1b4a	9.453<br>*9.116*	7.924<br>*7.973*	7.697<br>*6.238*
26	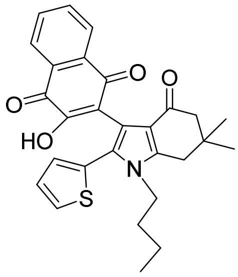	1d2c1b4b	8.706<br>*8.958*	8.045<br>*8.140*	8.349<br>*7.112*
27	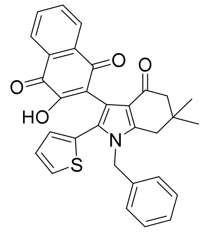	1d2c1b4g	9.149<br>*9.125*	8.344<br>*8.490*	8.024<br>*7.047*
28	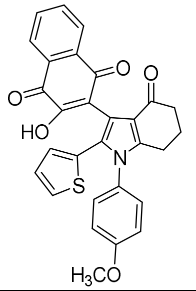	1d2c1c4a	9.008<br>*9.096*	7.727<br>*7.710*	7.086<br>*6.199*

29	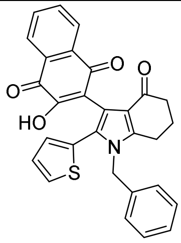	1d2c1c4g	8.687<br>*8.965*	8.238<br>*8.384*	7.247<br>*6.742*
30	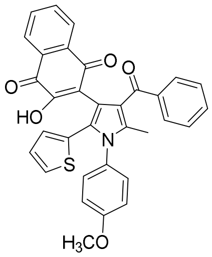	1d2c3b4a	9.030<br>*9.159*	7.962<br>*8.043*	7.383<br>*6.232*
31	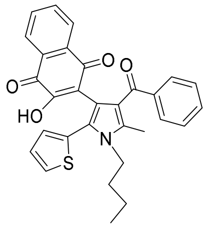	1d2c3b4b	8.308<br>*8.580*	7.946<br>*8.027*	7.955<br>*7.185*
32	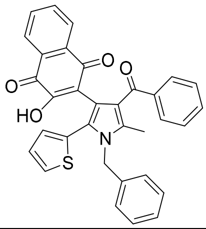	1d2c3b4g	8.798<br>*9.101*	8.399<br>*8.569*	7.904<br>*7.440*

Data are expressed in terms of the GA-MLR model (normal letter) and ANN model (italic letter) predicted percent scavenging activity (divided by 10) to [a] $^{\bullet}OH$ at 80μM, [b] $O_2^{\bullet -}$ at 20μM and [c] $DPPH^{\bullet}$ at 90μM of the designed compounds **16-32**.

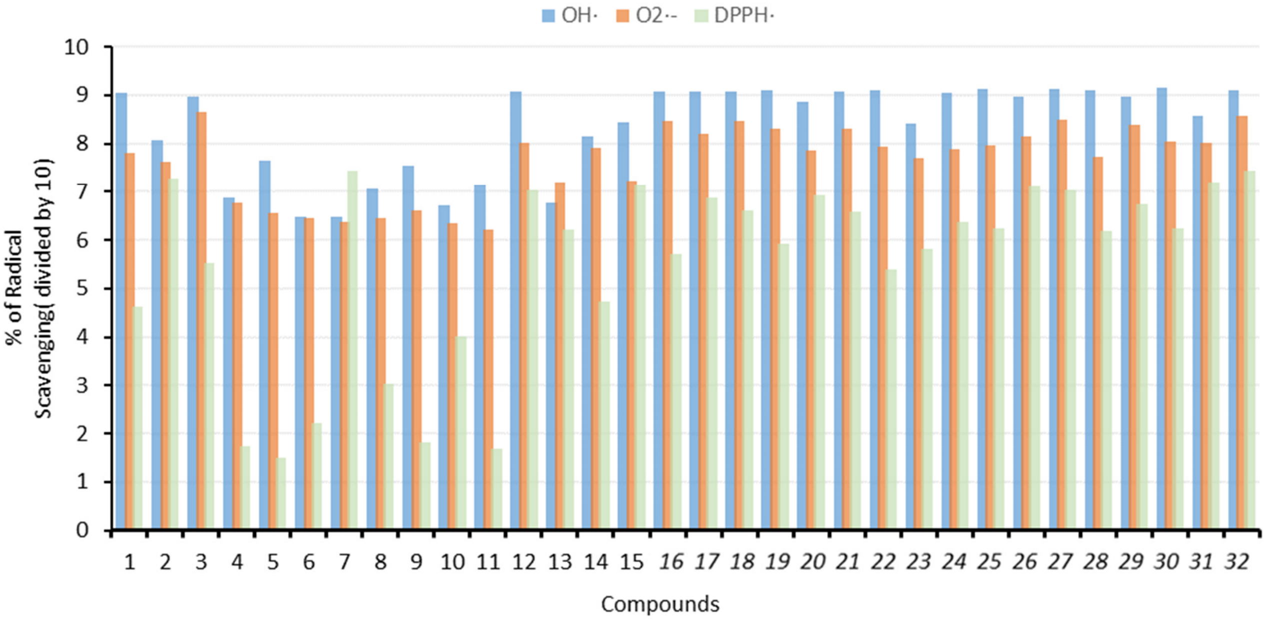

Figure S1. Experimental activities of previous synthesized compounds (**1-15**) and predicted antioxidant activities of newly designed compounds (***16-32***) by ANN models.

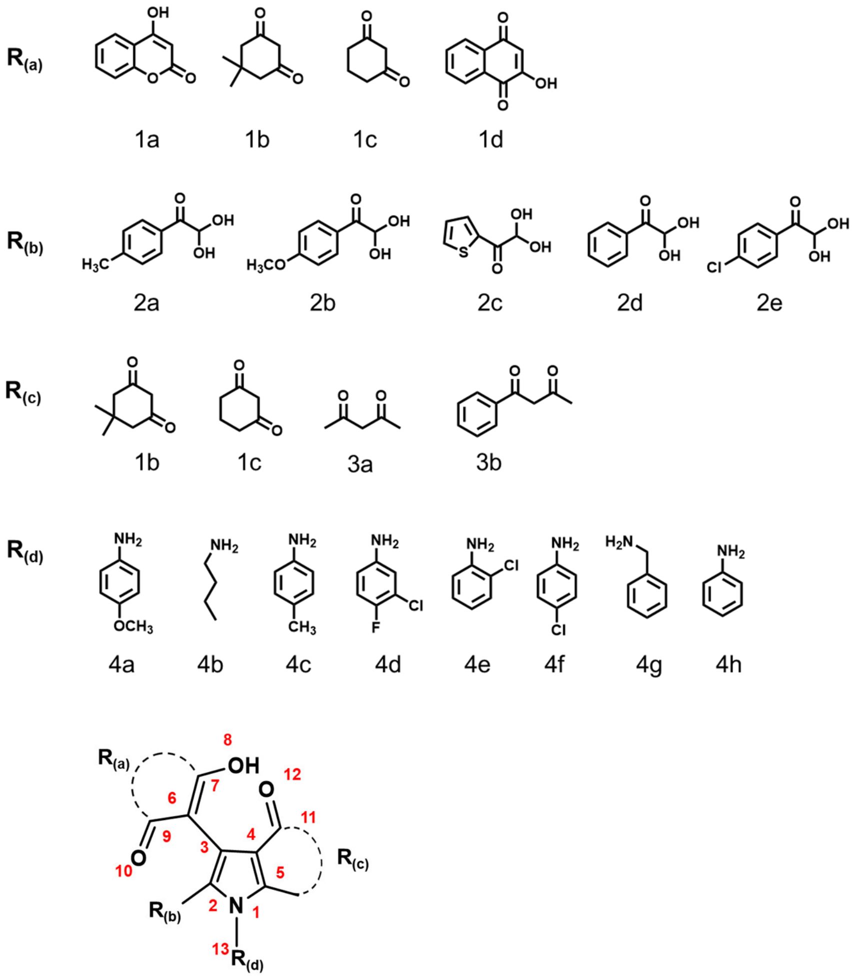

Figure S2. Starting reagents with codes and pyrrole derivatives' skeleton diagram with atom labels.

Table S4. 15 Pyrrole derivatives and their radical scavenging activities.

Cpd.	Structure	Code*	$^{\cdot}OH$ activity [a]	$O_2^{\cdot -}$ activity [b]	$DPPH^{\cdot}$ activity [c]
1	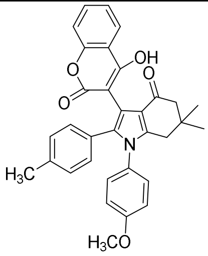	1a2a1b4a	9.047	7.811	4.627
2	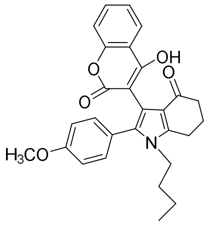	1a2b1c4b	8.010	7.622	7.126
3	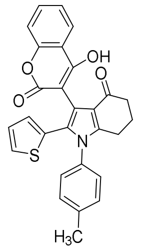	1a2c1c4c	8.991	8.644	5.517
4	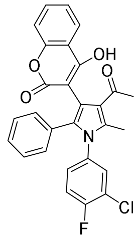	1a2d3a4d	6.848	6.781	1.772
5	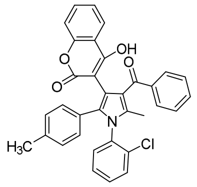	1a2a3b4e	7.588	6.485	1.561
6	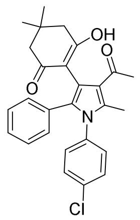	1b2d3a4f	6.365	6.498	2.504

7	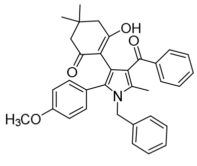	1b2b3b4g	6.658	6.341	7.604
8	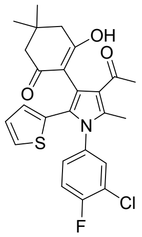	1b2c3a4d	7.094	6.443	3.211
9	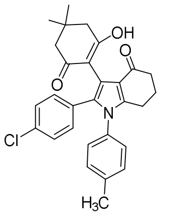	1b2e1c4c	7.388	6.578	1.747
10	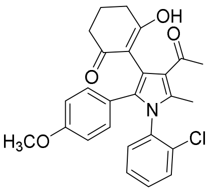	1c2a3a4e	6.681	6.371	3.889
11	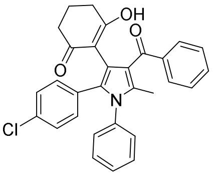	1c2e3b4h	7.294	6.203	1.348
12	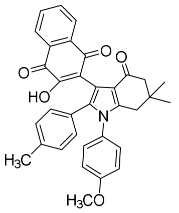	1d2a1b4a	9.151	8.057	7.044
13	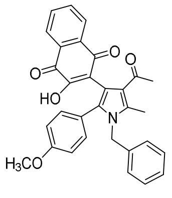	1d2b3a4g	6.757	7.209	6.391

14	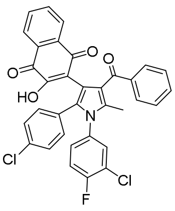	1d2e3b4d	8.148	7.832	4.745
15	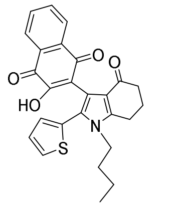	1d2c1c4b	8.431	7.264	6.941

Data are expressed in terms of the [percent scavenging activity/10] to [a] ·OH at 80µM, [b] $O_2^{\cdot -}$ at 20µM and [c] DPPH· at 90µM. * See code details in Figure S2.
